# Arachidonic acid downregulates acyl-CoA synthetase 4 expression by promoting its ubiquitination and proteasomal degradation[S]

**DOI:** 10.1194/jlr.M045971

**Published:** 2014-08

**Authors:** Chin Fung Kelvin Kan, Amar Bahadur Singh, Diana M. Stafforini, Salman Azhar, Jingwen Liu

**Affiliations:** * Department of Veterans Affairs Palo Alto Health Care System, Palo Alto, CA 94304; † Huntsman Cancer Institute, University of Utah, Salt Lake City, UT 84112

**Keywords:** posttranslational regulation, proteasome, nonalcoholic fatty liver disease

## Abstract

ACSL4 is a member of the long-chain acyl-CoA synthetase (ACSL) family with a marked preference for arachidonic acid (AA) as its substrate. Although an association between elevated levels of ACSL4 and hepatosteatosis has been reported, the function of ACSL4 in hepatic FA metabolism and the regulation of its functional expression in the liver remain poorly defined. Here we provide evidence that AA selectively downregulates ACSL4 protein expression in hepatic cells. AA treatment decreased the half-life of ACSL4 protein in HepG2 cells by approximately 4-fold (from 17.3 ± 1.8 h to 4.2 ± 0.4 h) without causing apoptosis. The inhibitory action of AA on ACSL4 protein stability could not be prevented by rosiglitazone or inhibitors that interfere with the cellular pathways involved in AA metabolism to biologically active compounds. In contrast, treatment of cells with inhibitors specific for the proteasomal degradation pathway largely prevented the AA-induced ACSL4 degradation. We further show that ACSL4 is intrinsically ubiquitinated and that AA treatment can enhance its ubiquitination. Collectively, our studies have identified a novel substrate-induced posttranslational regulatory mechanism by which AA downregulates ACSL4 protein expression in hepatic cells.

The liver plays a central role in the control of whole-body lipid metabolism by regulating the uptake, synthesis, oxidation, and export of FAs (in the form of VLDL-TG) to adapt to the needs of the organism under different nutritional conditions. Dysregulation of FA metabolism is now increasingly recognized as a contributing factor to the pathogenesis of nonalcoholic fatty liver disease (NAFLD), type 2 diabetes, insulin resistance, and metabolic syndrome (1). In order for FAs to enter biologically active pools, they must first be activated by acyl-CoA synthetases, which generate fatty acyl-CoA. Long-chain acyl-CoA synthetase (ACSL) catalyzes the formation of fatty acyl-CoAs from ATP, CoA, and long-chain FAs (carbon chain lengths of 12–20). Once formed, fatty acyl-CoAs can be metabolized through different metabolic pathways, including the cellular β-oxidation system responsible for FA oxidation (catabolism), and the anabolic pathways for the synthesis of phospholipids, cholesterol esters, and TG (2–4).

To date, five isoforms of ACSLs (ACSL1, ACSL3, ACSL4, ACSL5, and ACSL6) have been identified and characterized in human, mouse, and rat tissues (5). Although these isoforms catalyze similar enzymatic reactions, they exhibit variable cellular functions and generate distinct metabolic outcomes in an isoform-specific and tissue/cell type-specific manner. The current hypothesis is that substrate specificity, subcellular localization, tissue-specific expression, and upstream signaling regulatory pathways all contribute to the unique functions of the individual ACSLs.

Arachidonic acid (AA) (20:4, n-6) is an essential PUFA. It is a substrate for an important class of lipid mediators, eicosanoids, such as prostaglandins, thromboxanes, and leukotrienes (6). AA transported into cells from the exogenous sources or released from the endogenous sources, is rapidly converted to AA-CoA ester by acyl-CoA synthetases. Among various ACSLs, ACSL4 has a marked preference for AA and as such plays a major role in the cellular metabolism of AA (5).

In adult human tissues, the ACSL4 expression is detected at very high levels in brain, placenta, testis, ovary, spleen, and adrenal cortex; low levels are detected in liver (7, 8). However, in contrast to normal liver tissues, high levels of hepatic ACSL4 are detected in pathological conditions such as hepatocarcinoma (9) and NAFLD (10–12). Although studies have shown that ACSL4 exhibits proliferating properties to promote tumor growth and survival of hepatocaricinoma cells, currently, there is little information available about the specific role played by ACSL4 in hepatic lipid metabolism both under normal physiological conditions and in NAFLD. Moreover, the cellular mechanisms that regulate ACSL4 expression under normal physiological conditions and in the disease state remain largely unknown.

In the present study, we first examined the ACSL4 expression in liver tissues of mice fed a normal chow diet (NCD) or a high-fat diet (HFD). We unexpectedly observed that expression of ACSL4 protein was greatly reduced in response to feeding a HFD as compared with the control diet. Further exploration of this novel observation led to the identification of a unique posttranslational mechanism involved in the AA regulation of ACSL4, i.e., AA regulates ACSL4 protein by promoting its degradation via the ubiquitin (Ubq)-proteasome system. This regulatory mechanism is highly specific for both ACSL4 and AA; ACSL4 protein stability is not impacted by other saturated or unsaturated FAs, and AA exerts no significant effect on the protein levels of other ACSLs.

## MATERIALS AND METHODS

### Reagents

FA-free BSA, palmitic acid (PA), oleic acid (OA), AA, EPA, rosiglitazone, bortezomib, MG132, bafilomycin A1, and inhibitors to CYP2C and 2J (danazol and gemfibrozil) were purchased from Sigma. [9,10-^3^H(N)]PA (30–60 Ci; 1.11–2.22 TBq/mmol), [9,10-^3^H(N)]OA (15–60 Ci; 0.555–2.22 TBq/mmol), and [5,6,8,9,11,12,14,15-3H(N)]AA were obtained from Perkin-Elmer (Waltham, MA). Selective inhibitors of COX1 (SC-560), COX2 (CAY10404), and nonselective COX inhibitors (indomethacin and aspirin), 12- and 5-LOX inhibitor (3,4-dihydroxyphenyl ethanol, 5-LOX inhibitor (Zileuton), 15-LPX inhibitor 1, CYP4A, CYP4F inhibitor (HET0016), CYP2C9, 2C19, 3A inhibitor (fluconazole), and PGE2 were obtained from Cayman Chemicals (Ann Arbor, MI). Specific kinase inhibitors were purchased from Calbiochem (Temecula, CA). All other reagents used were of analytical grade.

### Animals and diets

All animal experiments were performed according to procedures approved by the Veterans Affairs Palo Alto Health Care System Animal Care and Use Committee. Seven-week-old male control C57BL/6J mice were obtained from the Jackson Laboratory (Bar Harbor, ME) and were fed either a HFD (#TD_·_06414; approximately 60% of total calories derived from fat, Harlan Laboratories), or a rodent NCD for 16 weeks. The mice fed the HFD developed obesity, mild to moderate hyperglycemia, hyperinsulinemia, type 2 diabetes, and steatosis as previously reported (13).

### Cell lines

HepG2 cells were obtained from ATCC. The HEK293A cell line was obtained from Invitrogen. Mouse primary hepatocytes were isolated from male C57BL/6J mice at San Francisco General Hospital Liver Center and were cultured as we previously described (14). HepG2 and Huh7 human hepatic cells were cultured in MEM with 10% FBS.

### Construction of pShuttle-ACSL4

The human ACSL4 coding region was amplified from a HepG2 cDNA library, cloned into pcDNA4.0-HisMax-TOPO vector to create pHis-ACSL4 plasmid. The ACSL4 coding sequence was subcloned from pHis-ACSL4 into the *Sal*I and *Xho*I sites of a pShuttle-IRES-hrGFP-1 vector that contained three contiguous copies of the Flag epitope (Stratagene, CA) to yield the resulting shuttle vector, pShuttle-ACSL4.

### Construction of human ACSL4 promoter and 3′UTR luciferase reporters

For generation of ACSL4 promoter reporter, a DNA fragment of 2,720 bp covering human ACSL4 proximal promoter region from −2,651 to +69 relative to the 5′end of exon 1 was amplified from HepG2 genomic DNA and was cloned into Topo 2.1 vector, followed by subcloning into pGL3-basic at the Sac1 and Xho1 sites to yield the promoter reporter pGL3-ACSL4. To construct ACSL4 3′untranslated region (UTR) reporter, a DNA fragment of 2,780 bp covering human ACSL4 v1 cDNA sequence from 2,260 to 5,039 and containing the entire 3′UTR was cloned into pcDNA-luciferase (Luc) vector (15) at the 3′end of the Luc coding sequence. After transformation and propagation in *Escherichia coli*, two independent clones per reporter were sequenced to verify the sequence and orientation of the promoter/3′UTR fragment.

### Luc reporter assay

Luc reporter assay was performed by using the Dual Luciferase Reporter Assay system (Promega) according to the manufacturer’s instructions. Two independent pGL3-ACSL4 plasmids or pcDNA-Luc-ACSL4-UTR plasmids were individually transfected into HepG2 cells along with plasmid pRL-SV40, a Renilla Luc reporter, as an internal transfection efficiency control. One day after transfection, cells were treated with the indicated doses of AA or the vehicle. Cells were lysed with 50 μl of lysis buffer followed by measurements of firefly Luc and Renilla Luc activities. The firefly Luc activity was normalized to Renilla activity. Four wells were assayed for each condition.

### RNA isolation and real-time quantitative RT-PCR

Total RNA was extracted from liver tissues using the Quick RNA Mini Prep kit (Zymo Research) and was reverse-transcribed into cDNA as described previously (16). Real-time quantitative RT-PCR (qRT-PCR) was performed with cDNA template and specific primers using a SYBR green PCR kit (Power SYBR® Green PCR Master Mix) and an ABI Prism 7700 system (Applied Biosystems® Life Technologies) according to the manufacturer’s protocols. qRT-PCR primers for each gene are listed in supplementary Table I. Target mRNA expression in each sample was normalized to the housekeeping gene, GAPDH. The 2^−ΔΔCt^ method was used to calculate relative mRNA expression levels.

### ACSL4 mRNA half-life determination

To determine ACSL4 mRNA half-life, HepG2 cells were treated with 50 μM of AA or vehicle for 8 h. Actinomycin D (5 μg/ml) was added to cells at different intervals (0, 1, 2, 4, 6, and 8 h). At the end of incubation, total RNA was extracted from the cells.

### Western blotting of ACSLs in mouse liver tissue and hepatic cells

Approximately 90–100 mg of frozen liver tissue from each mouse was homogenized in 1 ml RIPA buffer [50 mM Tris, 150 mM NaCl, 1 mM EDTA, 1% Triton X-100, 0.5% sodium deoxycholate, and 0.1% SDS (pH 7.4)] containing 1 mM PMSF and protease inhibitor cocktail (Roche). After protein quantification using BCA^TM^ protein assay reagent (Pierce), 100 μg of homogenate proteins from individual liver samples or 30 μg protein of total cell lysates from cell lines were separated on SDS-PAGE, transferred to nitrocellulose membranes, and detected using ACSL isoform-specific antibodies. Anti-human ACSL4 antibody was provided by Dr. Stephen Prescott (University of Utah, Salt Lake, UT) through his colleague, Dr. Diana Stafforini (Huntsman Cancer Institute, University of Utah) (8). The anti-ACSL4 antibody recognizes a 14 AA peptide of N-terminal sequences of ACSL4 of human, mouse, rat, and hamster origins (supplementary Fig. IA). Rabbit anti-hamster ACSL3 antibody was previously generated in our laboratory (17) that recognizes the C-terminal sequences of ACSL3 of human, mouse, rat, and hamster origins (supplementary Fig. IB). The anti-human ACSL1 (ab76702) and anti-human ACSL5 (ab104892) were obtained from Abcam, Cambridge, MA. The membranes were reprobed with an anti-β-actin (Sigma) or anti-GAPDH (Sigma). Immunoreactive bands of predicted molecular mass were visualized using an ECL Plus kit (GE Healthcare Life Sciences, Piscataway, NJ) and quantified with the Alpha View software with normalization by signals of β-actin or GAPDH.

### ACSL4 protein half-life determination

HepG2 cells were treated with 50 μM of AA or vehicle for 8 h. Cycloheximide (CHX) (5 μg/ml) was added for different times (0, 1, 2, 4, 6, and 8 h) and total cell lysates were harvested at the indicated times. Thirty micrograms of protein samples were subjected to SDS-PAGE followed by Western blotting for ACSL4 protein. To determine the half-life of ACSL4, CHX-treated cells were immunoblotted for ACSL4 and β-actin and quantitated. For each experiment, after normalization, the amount of ACSL4 at *t* = 0 was set to 100, the signal at different time points was plotted against time, and fitted to an exponential decay curve and the half-life (T_1/2_) was calculated using GraphPad Prism 5 software.

### Ubiquitination assay

Plasmids expressing HA-tagged Ubq (HA-Ubq) or Flag-tagged human ACSL4 (pShuttle-ACSL4) were cotransfected into HEK293A cells. Mock transfections with empty vectors were performed in parallel as control. At 48 h after transfection, cells were treated with 20 μM of the proteasomal inhibitor MG132 for 6 h before cell lysis. Then anti-HA or anti-Flag precipitates from the cell lysates were analyzed by Western blotting using anti-HA, anti-Flag, and anti-ACSL4 antibodies.

### Detection of endogenously ubiquitinated ACSL4 in HepG2 cells

HepG2 cells were treated for 8 h with 50 μM AA or control in the presence or absence of proteasome inhibitor MG132 (20 μM). Cells were lysed by addition of modified RIPA buffer [50 mM Tris (pH 7.4); NP-40, 1%; Na-deoxycholate, 0.25%; NaCl, 150 mM; and EDTA, 1 mM]. Cell lysates (0.5 ml) containing 600 μg protein were incubated with anti-ACSL4 antibody or a control antibody (rabbit IgG) overnight at 4°C with slow mixing. Protein A-agarose (Millipore) beads were added to the samples for another 3 h under continuous mixing. After incubation, the beads were collected by centrifugation and washed three times with modified RIPA buffer. All proteins were released from the agarose beads by boiling in 20 μl of 1× Laemmli sample buffer and then subjected to SDS-PAGE and Western blotting using anti-Ubq or anti-ACSL4 antibodies.

### Cell viability assay

Cells were seeded in a 96-well plate the day before treatment and treated for 24, 48, or 72 h with different concentrations of AA. The cell viability was measured using the CellTiter-Glo luminescent cell viability assay kit from Promega according to the manufacturer’s instructions. Four wells were evaluated under each experimental condition. In addition, a MTT-based colorimetric assay for quantification of cell proliferation and viability was conducted using Cell Proliferation Kit I (MTT) purchased from Roche.

### Measurement of ACSL activity

HepG2 cells were homogenized on ice in a buffer containing 20 mM HEPES, 1 mM EDTA, and 250 mM sucrose (pH 7.4). After a centrifugation at 16,000 rpm, cell lysates were collected and protein concentrations of cell lysates were determined by the BCA method (Pierce) and aliquots were stored at −80°C until assayed for ACSL activity. The incubation mixture contained 175 mM Tris-HCl (pH 7.4), 8 mM MgCl_2_, 5 mM dithiothreitol, 1 mM ATP, 0.2 mM CoASH, 0.5 mM Triton X-100, 10 μM EDTA, and 50 μM palmitate mixed with 0.1 μCi of [^3^H]PA, 0.1 μCi of [^3^H]OA, or 0.1 μCi of [^3^H]AA (18). The reaction was initiated by the addition of 4–5 μg protein, followed by incubation at room temperature for 20 min. The reaction was terminated by the addition of 1 ml Dole’s reagent (isopropanol: heptane:1 M H_2_SO_4_. 40:10:1). After two washes, radioactivity in the lower phase containing labeled [^3^H]acyl-CoA were measured by scintillation counting.

### FA loading of the cells

FA stock solution of 4.6 mM of PA, OA, AA, or EPA was made in heated (55°C) distilled water and subsequently added to 5% FA-free BSA for conjugation. The conjugated FA was applied to cells that were cultured in medium containing 10% FBS. Additionally, individual FAs were dissolved in DMSO to make a FA stock solution of 200 mM. FAs were added to the culture medium as the conjugated complex form of FA-free BSA (2:1 molar ratio). Cells were incubated in medium containing 10% FBS overnight prior to the addition of FA for the indicated time or concentration.

### Statistical analysis

Values are presented as mean ± SEM. Significant differences between diet groups and control and treatment groups were assessed by either one-way ANOVA with Bonferroni’s multiple comparison test or Student’s *t*-test. Statistical significance is displayed as *P* < 0.05 (one asterisk), *P* < 0.01 (two asterisks), or *P* < 0.001 (three asterisks).

## RESULTS

### Feeding a HFD downregulates the hepatic expression of ACSL4

**Fig. 1.**
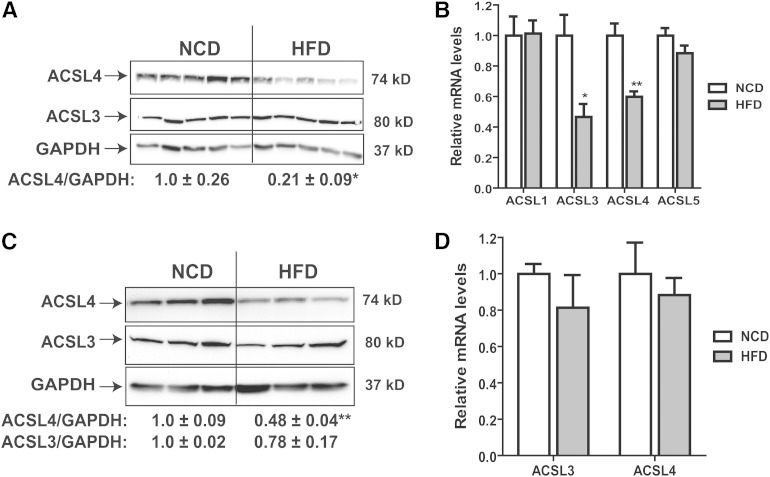
Downregulation of hepatic ACSL4 expression in vivo by feeding a HFD to mice. A, B: C57BL/6J male mice were fed a HFD or NCD (n = 5 per group) for 16 weeks. A: Homogenate proteins (100 μg) from individual liver samples were resolved by SDS-PAGE. Expression levels of different ACSL isoforms were detected by immunoblotting using isoform-specific antibodies. The protein amount of each ACSL isoform was quantified with the Alpha View software with normalization by signals of GAPDH. Values are mean ± SEM of five samples per group. B: Individual levels of ACSL mRNAs in liver samples of NCD and HFD mice were assessed by real-time qRT-PCR. After normalization with GAPDH mRNA levels, the relative mRNA level in the NCD group is expressed as 1. The results presented are mean ± SEM of five mice per group. C, D: In a second diet study, C57BL/6J male mice were fed a HFD or NCD (n = 3 per group) for 16 weeks. ACSL4 and ACSL3 protein and mRNA levels in the NCD and HFD groups were analyzed as in (A) and (B). The results presented are the mean ± SEM of three mice per groupQ11. * *P* < 0.05, ** *P* < 0.01, *** *P* < 0.001.

First, the mRNA and protein levels of ACSL4 in liver tissues from mice that were fed a HFD or a NCD for 16 weeks were measured. **Figure 1A** shows that HFD feeding markedly reduced the ACSL4 protein levels (∼80%; *P* < 0.05) in livers of HFD mice as compared with control (NCD) mice. Utilizing a highly specific anti-hamster ACSL3 antibody (17), we showed that in contrast to ACSL4, ACSL3 protein levels remained unchanged in response to HFD feeding. qRT-PCR analyses of four hepatic ACSL isoforms showed that ACSL4 mRNA levels were 40% lower in the HFD group as compared with the NCD group. The mRNA levels of ACSL1 and ACSL5 were unchanged, while ACSL3 mRNA levels were reduced by 50% upon HFD feeding (Fig. 1B) despite the unchanged ACSL3 protein levels. Given a reported association between elevated ACSL4 gene expression and human NAFLD (10), the observed lower levels ACSL4 protein in steatotic liver of HFD mice was unexpected. To confirm this finding, we analyzed liver samples of HFD and NCD mice from another diet experiment. We observed that ACSL4 protein levels in HFD group were 52% lower than those of the NCD group (*P* < 0.01), while ACSL4 mRNA levels remained unchanged (Fig. 1C, D). Thus, the mean reduction of ACSL4 protein by HFD feeding from the two separate diet studies was approximately 65%.

**Fig. 2.**
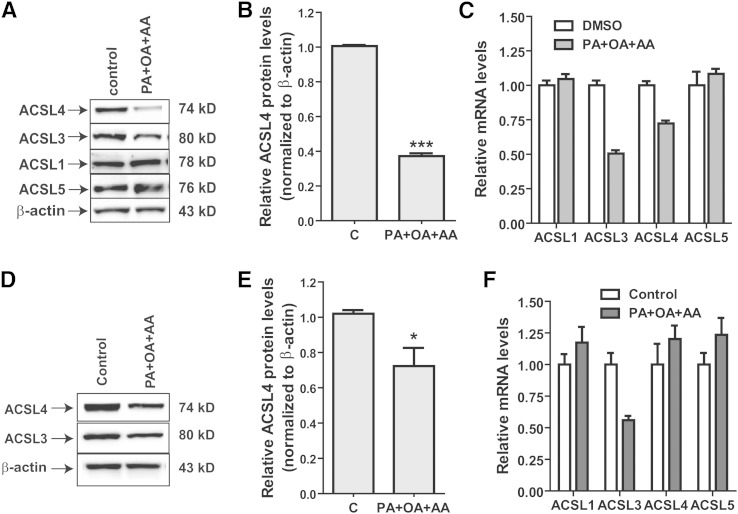
Downregulation of hepatic ACSL4 expression in hepatic cells by FAs. A–D: HepG2 or Huh7 cells were treated with a mixture of FAs containing 65 μM PA, 65 μM OA, and 20 μM AA. After 24 h of FA treatment, total cell lysates were isolated for Western blotting and total RNA was isolated for qRT-PCR. After Western blotting, the protein amount of each ACSL isoform was quantified with the Alpha View software with normalization by signals of β-actin. The data are the mean ± SEM of three independent experiments. * *P* < 0.05, *** *P* < 0.001. C, control.

To investigate the underlying mechanism involved in the repression of ACSL4 expression in response to HFD feeding of mice, we attempted to mimic the hyperlipidemic conditions in vitro by culturing hepatic cell lines in medium supplemented with a mixture of saturated (PA), monounsaturated (OA), and polyunsaturated (AA) FAs. Exposure of cells to FAs reduced the ACSL4 protein levels to 37% of control (*P* < 0.001) in HepG2 cells (**Fig. 2A, B**) and to 72% of control (*P* < 0.05) in Huh7 cells (Fig. 2D, E). Again, the expression of ACSL1 or ACSL5 protein was not affected by such manipulation, and ACSL3 protein levels were slightly decreased in HepG2 and Huh7 cells. Furthermore, despite the significant decreases in ACSL4 protein levels, ACSL4 mRNA levels were only minimally impacted by FA treatment in HepG2 cells and unaffected in Huh7 cells (Fig. 2C, F). Altogether, these in vivo and in vitro results suggest that exposure of liver cells to excessive amounts of FAs downregulates ACSL4 expression mainly at the protein level.

### ACSL4 protein is specifically downregulated by its preferred substrate, AA

**Fig. 3.**
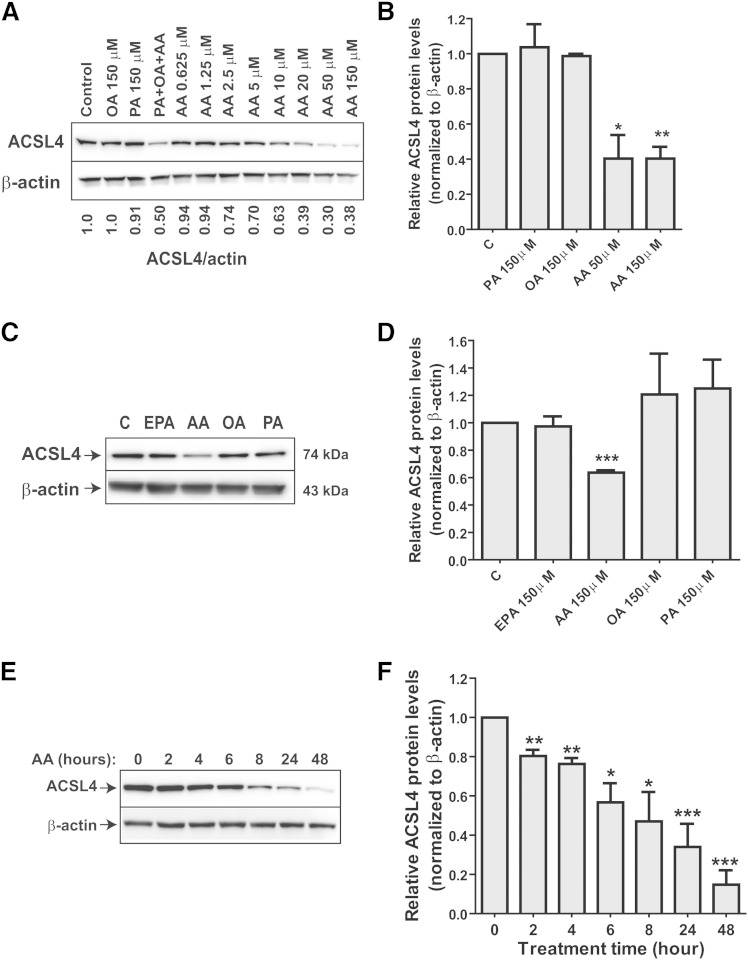
Dose- and time-dependent downregulation of ACSL4 protein by AA. A, B: HepG2 cells were incubated for 24 h with the indicated concentrations of exogenous FA. ACSL4 protein levels were detected by Western blotting. After Western blotting, the protein amount of ACSL4 was quantified with the Alpha View software with normalization by signals of β-actin. The data in (A) are representative of three separate experiments. The indicated value is for the blot shown. The data presented in (B) are the mean ± SEM of three independent treatment experiments. C, D: Mouse primary hepatocytes were treated with 150 μM of each FA for 24 h. ACSL4 protein levels were detected by Western blotting. The data presented in (D) are the mean ± SEM of three independent treatment experiments. E, F: ACSL4 protein levels were examined in HepG2 cells that were treated with 150 μM AA for the indicated times. The Western blotting result shown in (E) is representative of three separate assays, and the quantitative results are presented as the mean ± SEM of three independent kinetic studies. C, control.

To identify specific FA species that affect ACSL4 protein expression, we treated HepG2 cells with the indicated concentrations of individual FAs of varying chain length and degree of saturation (19). PA or OA exposure of cells did not produce a suppressive effect on ACSL4 protein levels, whereas inclusion of AA in the culture medium greatly suppressed the ACSL4 protein abundance in a dose-dependent manner. **Figure 3A** is a representative Western blot analysis. Quantitative data presented in Fig. 3B are derived from three independent experiments. These data show that a 5 μM concentration of AA caused lowering of ACSL4 protein levels by ∼30%, while a maximum reduction in ACSL4 protein levels was achieved at an AA concentration of 50 μM. None of the doses of AA applied had any significant effect on cell viability (supplementary Fig. IIA). Furthermore, ACSL4 mRNA levels were not reduced over the AA concentration range (supplementary Fig. IIB). In contrast, SREBP1c mRNA levels were dose-dependently lowered by AA treatment in HepG2 cells, which was in line with literature reports (20). We also measured acyl-CoA synthesis activity and showed that treatment of HepG2 cells with AA (150 μM) for 24 h reduced the arachidonoyl-CoA synthetase activity by 46% (*P* < 0.01) (supplementary Fig. IIC). The specific inhibitory effect of AA on ACSL4 protein expression was also detected in mouse primary hepatocytes (Fig. 3C, D).

To examine the kinetics of AA action on ACSL4 protein expression, we treated HepG2 cells with a saturating concentration of AA (150 μM) for various time points. Figure 3E is a representative Western blot analysis. Quantitative data presented in Fig. 3F are derived from three independent kinetics experiments which indicate that AA can lower ACSL4 protein levels in a relatively short time; following AA exposure, ACSL4 protein levels were reduced by 53% at 8 h (*P* < 0.05) and by 66% (*P* < 0.001) at 24 h, as compared with the vehicle control.

### AA treatment reduces ACSL4 protein half-life without affecting gene transcription or the mRNA stability

**Fig. 4.**
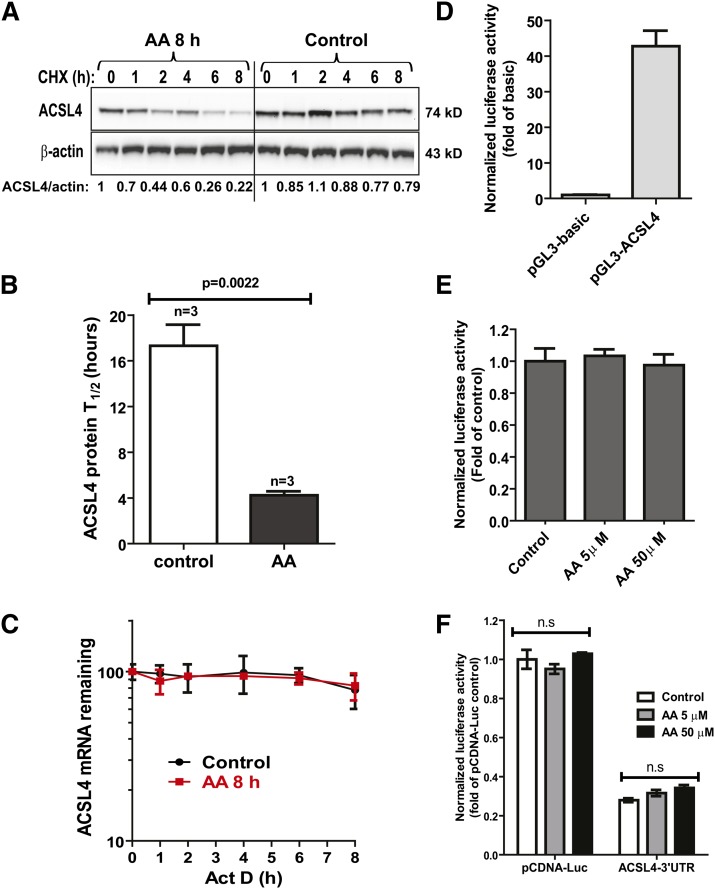
AA reduces ACSL4 protein half-life without affecting ACSL4 gene transcription or mRNA stability. A: HepG2 cells were treated with 50 μM AA or vehicle for 8 h. CHX, at 5 μg/ml concentration, was added to cells for the indicated times. Total cell lysates were subjected to Western blotting and bands were visualized with antibody against ACSL4 or β-actin. The indicated value is for the blot shown. B: After normalization to β-actin, the ACSL4 signal intensity was plotted against the CHX treatment time to calculate T_1/2_ of ACSL4 protein. The T_1/2_ data presented are the mean ± SEM of three independent CHX treatment experiments. C: HepG2 cells were treated with 50 μM AA or vehicle for 8 h. Then, actinomycin D (Act D), at a concentration of 5 μg/ml, was added to the cells and total RNA was isolated at the indicated treatment times for qRT-PCR analysis of ACSL4 and GAPDH. After normalization with GAPDH, ACSL4 mRNA levels were plotted against the treatment time. D: Reporter constructs were cotransfected with a Renilla expression vector (pRL-SV40) into HepG2 cells. Two days post transfection, cell lysates were isolated to measure dual Luc activities. After normalization, the Luc activity of pGL3-basic is expressed as 1 and the Luc activity of pGL3-ACSL4 is expressed as fold of pGL3-basic. E: HepG2 cells were transfected with pGL3-ACSL4 and pRL-SV40 for 2 days prior to AA treatment of 24 h at the indicated doses. Cell lysates were isolated to measure dual Luc activities. The normalized Luc activity in control cells is expressed as 1. F: HepG2 cells were transfected with pcDNA-Luc control vector or pcDNA-Luc-ACSL4 3′UTR plasmid (ACSL4-UTR) for 1 day prior to AA treatment of 24 h at the indicated doses. Cell lysates were isolated to measure dual Luc activities. The normalized Luc activity of pCDNA-Luc in control cells is expressed as 1. n.s., not significant.

To determine whether AA lowering of ACSL4 protein levels was due to its increased degradation, we treated HepG2 cells with or without AA and/or with or without a protein synthesis inhibitor, CHX, and changes in ACSL4 protein levels were followed for the next 8 h by the Western blotting. Three separate experiments with identical conditions were performed. **Figure 4A** is a representative Western blot analysis. Quantitative data presented in Fig. 4B are derived from three independent experiments. AA treatment caused an accelerated degradation of ACSL4 protein with T_1/2_ = 4.2 ± 0.35 h as compared with T_1/2_ = 17.3 ± 1.84 h (*P* < 0.01) calculated from non-AA-treated control cells.

We next assessed ACSL4 mRNA stability in control and AA-treated HepG2 cells by blocking new mRNA synthesis with a transcription inhibitor, actinomycin D. Figure 4C shows that AA treatment had no effect on the steady state levels of ACSL4 mRNA.

To further examine a possible effect of AA on ACSL4 gene transcription, we cloned a 2.7 kb fragment of the 5′ proximal promoter region of the human ACSL4 gene into a promoterless Luc reporter, pGL3-basic, and transfected reporter constructs into HepG2 cells followed by treatment with or without low or high doses of AA. ACSL4 promoter activity was 40-fold of pGL3-basic and it was unaffected by treatment of cells with both low (5 μM) and high (50 μM) concentrations of AA (Fig. 4D, E).

The 3′UTRs of mRNAs play crucial roles in posttranscriptional regulation of gene expression by destabilizing mRNA or inhibiting protein synthesis (20), consequently, lowering protein levels. To determine if AA affects ACSL4 protein levels through its 3′UTR, we constructed a pcDNA-Luc-ACSL4-3′UTR reporter and measured the reporter Luc activity in response to AA treatment. Inclusion of the entire ACSL4 3′UTR lowered the Luc activity of the control vector pcDNA-Luc by 72%, but AA did not change the UTR reporter activity in HepG2 cells (Fig. 4F). Collectively, these data firmly established that AA downregulates ACSL4 expression by a posttranslational mechanism.

### AA downregulates ACSL4 protein levels independent of pathways involved in its cellular metabolism

It is well-known that oxidative metabolism of unesterified AA by cyclooxygenases and lipoxygenases is the major source for the production of biologically active eicosanoids (19). In addition, cytochrome P450 systems participate in free AA metabolism (10, 21). To assess the contribution of these pathways in AA regulation of ACSL4 protein levels, we treated HepG2 cells with various AA metabolic enzyme inhibitors for 1 h prior to AA addition for 24 h. We did not observe any effects of these inhibitors on AA-mediated degradation of ACSL4 protein by Western blotting. In addition, we treated HepG2 cells with rosiglitazone, a specific ACSL4 enzymatic inhibitor (22). We observed that rosiglitazone at 10 and 20 μM inhibited arachidonoyl-CoA synthetase activity. However, addition of this inhibitor to HepG2 cells did not prevent AA from downregulation of ACSL4 protein expression (supplementary Fig. IIIA, B), suggesting that the repressive effect of AA on ACSL4 protein level is independent of ACSL4 enzymatic activity. Furthermore, we tried specific inhibitors to several kinases that were previously implicated in the regulation of ACSL4 expression in other cell types (23). All inhibitor study results are summarized in supplementary Table II, which shows lack of effects of these inhibitors on AA-induced ACSL4 degradation.

### Involvement of the Ubq-proteasome pathway in AA-induced ACSL4 protein degradation

**Fig. 5.**
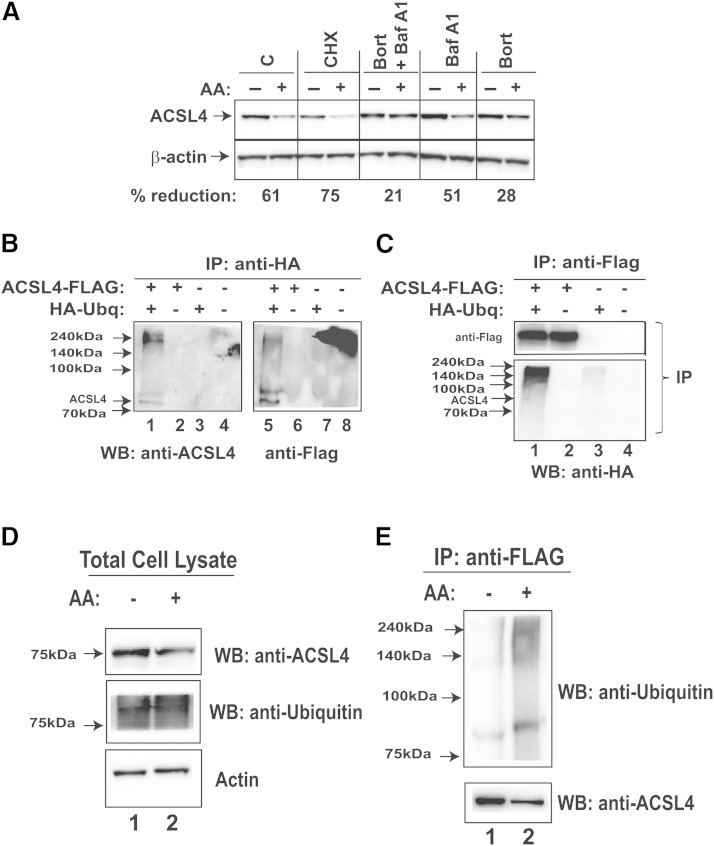
Proteasomal pathway participates in AA-induced degradation of ACSL4 protein. A: HepG2 cells were treated with 5 μg/ml CHX, 200 nM bortezomib (Bort), 50 nM bafilomycin A1 (Baf A1), or the combination of Bort and Baf A1 for 1 h prior to the addition of 150 μM AA. Cell lysates were isolated after 24 h of AA treatment. After Western blotting, for each sample, the signal of ACSL4 was normalized to signal of β-actin. The normalized ACSL4 signal without AA treatment was expressed as 100%. The data are representative of two separate experiments with similar results. The indicated value is for the blot shown. C, control. B, C: Plasmids encoding Flag-tagged ACSL4 and HA-Ubq were cotransfected into HEK293 cells. The empty vectors of pCMV-Entry and pCMV-HA were transfected as mock control. Two days post transfection, cells were treated with 20 μM MG132 to block proteasomal degradation for 6 h prior to cell lysis. Cell lysates were immunoprecipitated with anti-HA or anti-Flag antibodies, respectively. D, E: HEK293A cells were cotransfected with ACSL4-FLAG and HA-Ubq plasmids for 48 h. Then, cells were divided equally into two plates. After overnight culturing, cells were treated with 150 μM AA or vehicle for 8 h in the presence of MG132 before isolation of total cell lysates. Proteins (300 μg) from each lysate sample were subjected to IP with anti-FLAG. Total lysates were analyzed for ACSL4 and ubiquitinated proteins by immunoblotting using anti-ACSL4 and anti-Ubq antibody (D). IP complexes were analyzed for total ACSL4 with anti-ACSL4 antibody and ubiquitinated ACSL4 by anti-Ubq antibody (E). WB, Western blot.

The marked reduction in ACSL4 protein half-life in AA-treated cells suggested that ACSL4 is subject to a rapid degradation process. The autophagy-lysosomal pathway and the Ubq-proteasome pathway are the two major cellular proteolytic systems that participate in intracellular protein degradation in eukaryotic cells. To determine which of these two pathways is primarily involved in AA-induced ACSL4 degradation, we pretreated HepG2 cells with bafilomycin A1 (a specific lysosomal inhibitor) and bortezomib (a proteasomal inhibitor), either separately or in combination and subsequently exposed to AA or vehicle (control). The protein synthesis inhibitor, CHX, was used as a negative control in these experiments. **Figure 5A** shows that bortezomib alone or combined with bafilomycin A1 largely blocked the AA-mediated degradation of ACSL4. On the contrary and as expected, treatment of cells with CHX was without any effect. These data suggested that AA mainly utilizes the Ubq-proteasome pathway to promote ACSL4 degradation.

The degradation of a protein by the Ubq-proteasome system involves two distinct and successive steps: *1*) covalent attachment of multiple Ubq molecules to the target protein; and *2*) degradation of ubiquitinated protein by the 26S proteasome (24). To detect ubiquitinated ACSL4, first we constructed a plasmid vector (pShuttle-ACSL4) to express human ACSL4 with a Flag tag at the C terminus. Next, we cotransfected HEK293 cells with pShuttle-ACSL4 and a plasmid (HA-Ubq) expressing a HA-Ubq. In parallel, cells were transfected with respective empty vectors as negative controls. Whole cell lysates were subjected to immunoprecipitation (IP) with anti-Flag or anti-HA antibody conjugated to agarose beads. The anti-HA immunoprecipitates were subjected to SDS-PAGE followed by visualization of blots with anti-ACSL4 and anti-Flag antibodies. Both mono- and multiple ubiquitinated species of ACSL4 proteins were detected (Fig. 5B, lanes 1 and 5). Likewise, Western blotting of anti-Flag precipitates with anti-HA antibody demonstrated the presence of the mono- and polyubiquitinated ACSL4 in cell extracts that were derived from cells cotransfected with ACSL4-flag and HA-Ubq (Fig. 5C, lane 1). These data confirmed that ACSL4 is subject to ubiquitination.

Next, we addressed the question of whether AA induction of ACSL4 degradation is also accompanied by an enhanced ubiquitination of ACSL4. First, we cotransfected HEK293A cells with flag-tagged ACSL4 and HA-Ubq plasmids. Two days post transfection, cells were treated with AA or vehicle for 8 h. Equal amounts of whole cell lysates were subjected to IP with anti-FLAG beads followed by Western blotting using anti-ACSL4 or anti-Ubq antibody. Detection of ACSL4 and Ubq in total cell lysates showed that the AA treatment reduced the ACSL4 protein amount compared with the control, while both samples had similar cellular levels of ubiquitinated proteins (Fig. 5D). However, after FLAG IP, even though the amount of pulled down ACSL4 was lower in the precipitates of the AA-treated sample than that of the control sample, the AA-treated sample clearly had a higher amount of ubiquitinated ACSL4 than the control (Fig. 5E).

**Fig. 6.**
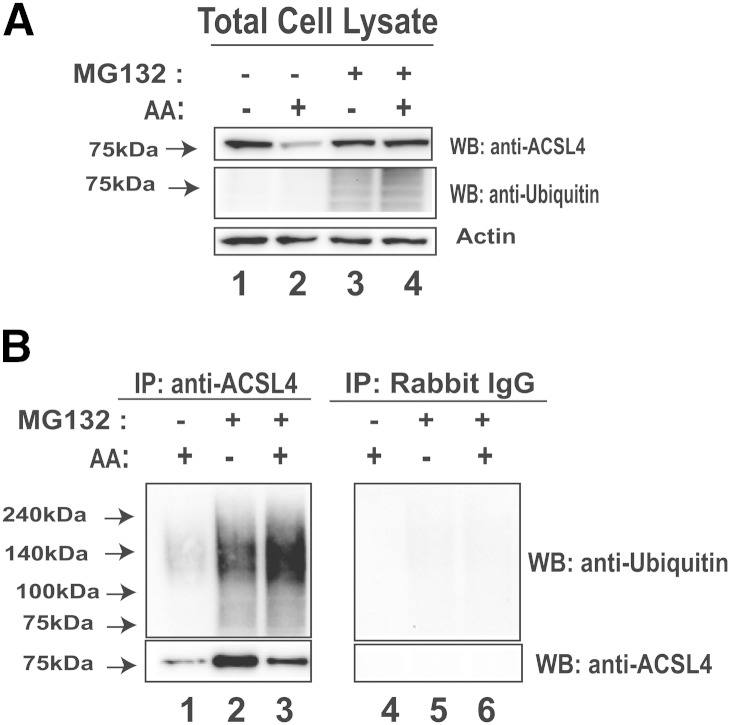
Detection of ubiquitination of endogenous ACSL4 in HepG2 cells without and with AA treatment. HepG2 cells were treated for 8 h with 50 μM AA or control in the presence or absence of proteasome inhibitor MG132 (20 μM). Proteins (600 μg) from each lysate sample were subjected to IP with anti-ACSL4 antibody or control antibody, rabbit IgG. A: Total lysates were analyzed for ACSL4 and ubiquitinated proteins by immunoblotting using anti-ACSL4 and anti-Ubq antibody. B: IP complexes were analyzed for ubiquitinated ACSL4 with anti-ACSL4 antibody and anti-Ubq antibody. WB, Western blot.

Finally, we examined the ubiquitination status of endogenous ACSL4 in HepG2 cells. HepG2 cells were treated for 8 h with 50 μM AA or control in the presence or absence of proteasome inhibitor MG132 (20 μM). Equal amounts of whole cell lysates were subjected to IP with anti-ACSL4 antibody or a control antibody (rabbit IgG), followed by Western blotting using anti-ACSL4 or anti-Ubq antibody. Detection of ACSL4 and Ubq in total cell lysates showed that the AA treatment reduced ACSL4 protein amount compared with the control, and this reduction was abolished by MG132 (**Fig. 6A**, compare lane 2 to lane 4). Cellular levels of ubiquitinated proteins were barely seen in the absence of MG132, but were readily detectable in the MG132-treated sample, and the signal intensity was slightly higher by cotreatment with AA (Fig. 6A, lane 3 vs. lane 4). Importantly, after ACSL4 IP, the amount of pulled down unubiquitinated ACSL4, as shown by anti-ACSL4 Western blot, was lower in the precipitates of AA-treated sample than that of the control sample (Fig. 6B, compare lane 3 with lane 2); however, the AA-treated sample clearly had a higher amount of polyubiquitinated ACSL4 than the untreated control. In contrast with anti-ACSL4 IP, Western blotting with anti-ACSL4 or anti-Ubq antibodies did not detect specific bands in control IgG immunoprecipitates (Fig. 6B, lanes 4 to 6). These data are highly consistent with the results obtained in ACSL4-overexpressing cells (Fig. 5). Altogether, these results provide direct evidence that AA exposure leads to enhanced ACSL4 ubiquitination and possibly channeling of ACSL4 toward its proteasomal degradation.

## DISCUSSION

The ligase family of ACSL plays a pivotal role in hepatic FA metabolism and is essentially involved in virtually every aspect of cellular utilization of FA, including as an energy source, as building blocks, and as signaling molecules. During the past decade, a great deal of progress has been made in our understanding of the transcriptional regulation of the ACSL family, both under normal physiological as well as pathophysiological conditions. At the gene transcription level, ACSL1 is regulated by the PPAR family members through a PPAR-responsive element embedded in the C-promoter region of the ACSL1 gene (25). The transcription of ACSL5 is positively regulated by SREBP1c (26), whereas ACSL3 gene expression is regulated by both PPAR (27) and liver X receptor transcription factors (16, 28). On the other hand, ACSL4 gene expression in mouse liver is regulated by the circadian clock gene (29) and by Sp1 and CREB (30) in mouse steroidogenic Leydig cells. At the posttranscriptional level, ACSL1 and ACSL4 mRNA levels are negatively regulated by miR-34 (31). Until this study, however, there was no information available about the posttranslational regulation of the ACSL family members. Of great significance, we now have identified a novel substrate-mediated posttranslational regulation of ACSL4 to suppress its protein levels through the Ubq-proteasomal pathway.

There are several important new findings that resulted from the current studies. First, among ACSL family members, AA treatment markedly and selectively reduced ACSL4 protein levels without affecting cellular levels of ACSL1 and ACSL5 in model hepatoma cell lines, including HepG2 and Huh7 cells as well as in primary mouse hepatocytes. Although, AA treatment caused a reduction in ACSL3 protein levels, the extent of lowering was less as compared with ACSL4 protein, and ACSL3 mRNA expression was reduced to a much greater extent compared with ACSL3 protein levels. Our further analysis of ACSL3 promoter activity confirmed that AA inhibits ACSL3 transcription in a liver X receptor element-dependent manner, which accounts for the lowered ACSL3 protein levels in AA-treated cells (unpublished observations). In contrast to ACSL3, AA markedly reduced ACSL4 protein half-life without affecting ACSL4 gene transcription or the stability of its mRNA, as demonstrated by the lack of AA effect on ACSL4 promoter activity or the ACSL4 mRNA 3′UTR reporter activity. Thus, AA regulates ACSL4 expression by specifically targeting ACSL4 protein levels and promoting its degradation.

By detection of ubiquitinated ACSL4 in HepG2 cells without and with AA treatment, our study provides strong evidence showing that ACSL4 is ubiquitinated and AA treatment enhances ACSL4 ubiquitination.

Second, although both EPA and AA serve as substrates for ACSL4 (5), concentrations of EPA (up to 150 μM) did not show significant effects in reducing ACSL4 protein levels in primary hepatocytes, as well as in model hepatic cell lines (data not shown). On the other hand concentration of AA (as low as 5 μM) caused a substantial reduction in ACSL4 protein levels.

Third, it has been reported that free AA induces apoptosis in colon cancer cells and overexpression of ACSL4 blocked AA-induced apoptosis (32). In our studies, no induction of apoptosis or alteration in cell viability was notable under the conditions in which AA treatment caused a marked reduction in ACSL4 protein levels.

Among the various long-chain FAs, AA occupies a unique position in cellular lipid metabolism. In addition to conjugation by acyl-CoA synthetases and subsequent fatty acyl-CoA metabolism via oxidation and lipogenesis, free AA can be metabolized by cyclooxygenases, lipoxygenase, and cytochrome p450 enzyme systems into important lipid mediators and signaling molecules. By employing specific inhibitors against these enzyme activities, we obtained evidence that these enzymatic pathways have no demonstrable role in AA-induced downregulation of ACSL4 protein in HepG2 cells.

While this work was in preparation, Klett et al. (33) reported that ACSL4 protein and mRNA can be downregulated by AA and LA (linoleic acid) in rat insulinoma cells, INS 832/13. Because intracellular LA is readily convertible to AA, it is likely that the modulatory action of LA on ACSL4 expression reported by Klett et al. may indeed occur through AA. Based on these findings, we speculate that the downregulation of ACSL4 protein in the liver of the HFD mice reported here is also achieved via the conversion of LA to AA. This is a highly likely possibility given that rodent HFD is enriched in this particular type of PUFA.

It is worthy to mention here that HFD-mediated reduction in hepatic ACSL4 protein levels in C57/BL6 mice occurs under conditions of excessive obesity, mild to moderate hyperglycemia, hyperinsulinemia, diabetes, and hepatic steatosis (13). Currently, it is not clear how deficiency in ACSL4 potentially contributes to hepatic steatosis in HFD mice or the pathogenesis of human NAFLD. Obviously, further work will be required to determine the underlying mechanism(s) by which ACSL4 participates in the development of these metabolic diseases. Moreover, further exploration of the role of ACSL4 in the pathophysiology of these metabolic diseases may identify novel targets that may be exploited in the development of new therapies to treat these clinical conditions.

In summary, our studies have identified a novel substrate-induced posttranslational regulatory mechanism by which AA selectively downregulates ACSL4 protein expression through the Ubq-proteasomal pathway.

## Supplementary Material

Supplemental Data

## References

[1] Scorletti E. Byrne C. D. 2013 Omega-3 fatty acids, hepatic lipid metabolism, and nonalcoholic fatty liver disease. Annu. Rev. Nutr. 33: 231–248. 23862644 10.1146/annurev-nutr-071812-161230

[2] Currie E. Schulze A. Zechner R. Walther T. C. Farese R. V. Jr 2013 Cellular fatty acid metabolism and cancer. Cell Metab. 18: 153–161. 23791484 10.1016/j.cmet.2013.05.017 PMC3742569

[3] Ellis J. M. Frahm J. L. Li L. O. Coleman R. A. 2010 Acyl-coenzyme A synthetases in metabolic control. Curr. Opin. Lipidol. 21: 212–217. 20480548 10.1097/mol.0b013e32833884bb PMC4040134

[4] Coleman R. A. Lewin T. M. Van Horn C. G. Gonzalez-Baró M. R. 2002 Do acyl-CoA synthetases regulate fatty acid entry into synthetic versus degradative pathways? J. Nutr. 132: 2123–2126. 12163649 10.1093/jn/132.8.2123

[5] Soupene E. Kuypers F. A. 2008 Mammalian long-chain acyl-CoA synthetases. Exp. Biol. Med. (Maywood). 233: 507–521. 18375835 10.3181/0710-MR-287 PMC3377585

[6] Astudillo A. M. Balgoma D. Balboa M. A. Balsinde J. 2012 Dynamics of arachidonic acid mobilization by inflammatory cells. Biochim. Biophys. Acta. 1821: 249–256. 22155285 10.1016/j.bbalip.2011.11.006

[7] Cao Y. Traer E. Zimmerman G. A. McIntyre T. M. Prescott S. M. 1998 Cloning, expression, and chromosomal localization of human long-chain fatty acid-CoA ligase 4 (FACL4). Genomics. 49: 327–330. 9598324 10.1006/geno.1998.5268

[8] Cao Y. Murphy K. J. McIntyre T. M. Zimmerman G. A. Prescott S. M. 2000 Expression of fatty acid-CoA ligase 4 during development and in brain. FEBS Lett. 467: 263–267. 10675551 10.1016/s0014-5793(00)01159-5

[9] Sung Y. K. Hwang S. Y. Park M. K. Bae H. I. Kim W. H. Kim J. C. Kim M. 2003 Fatty acid-CoA ligase 4 is overexpressed in human hepatocellular carcinoma. Cancer Sci. 94: 421–424. 12824887 10.1111/j.1349-7006.2003.tb01458.x PMC11160225

[10] Westerbacka J. Kolak M. Kiviluoto T. Arkkila P. Siren J. Hamsten A. Fisher R. M. Yki-Jarvinen H. 2007 Genes involved in fatty acid partitioning and binding, lipolysis, monocyte/macrophage recruitment, and inflammation are overexpressed in the human fatty liver of insulin-resistant subjects. Diabetes. 56: 2759–2765. 17704301 10.2337/db07-0156

[11] Kotronen A. Yki-Jarvinen H. Aminoff A. Bergholm R. Pietilainen K. H. Westerbacka J. Talmud P. J. Humphries S. E. Hamsten A. Isomaa B. 2009 Genetic variation in the ADIPOR2 gene is associated with liver fat content and its surrogate markers in three independent cohorts. Eur. J. Endocrinol. 160: 593–602. 19208777 10.1530/EJE-08-0900

[12] Stepanova M. Hossain N. Afendy A. Perry K. Goodman Z. D. Baranova A. Younossi Z. 2010 Hepatic gene expression of Caucasian and African-American patients with obesity-related non-alcoholic fatty liver disease. Obes. Surg. 20: 640–650. 20119733 10.1007/s11695-010-0078-2

[13] Zhang H. Shen W. J. Cortez Y. Kraemer F. B. Azhar S. 2013 Nordihydroguaiaretic acid improves metabolic dysregulation and aberrant hepatic lipid metabolism in mice by both PPARα-dependent and -independent pathways. Am. J. Physiol. Gastrointest. Liver Physiol. 304: G72–G86. 23104557 10.1152/ajpgi.00328.2012 PMC3543637

[14] Dong B. Wu M. Cao A. Li H. Liu J. 2011 Suppression of Idol expression is an additional mechanism underlying statin-induced up-regulation of hepatic LDL receptor expression. Int. J. Mol. Med. 27: 103–110. 21069265 10.3892/ijmm.2010.559

[15] Li H. Chen W. Zhou Y. Abidi P. Sharpe O. Robinson W. H. Liu J. 2009 Identification of mRNA-binding proteins that regulate the stability of LDL receptor mRNA through AU-rich elements. J. Lipid Res. 50: 820–831. 19141871 10.1194/jlr.M800375-JLR200 PMC2666168

[16] Dong B. Kan C. F. Singh A. B. Liu J. 2013 High-fructose diet downregulates long-chain acyl-CoA synthetase 3 expression in liver of hamsters via impairing LXR/RXR signaling pathway. J. Lipid Res. 54: 1241–1254. 23427282 10.1194/jlr.M032599 PMC3622321

[17] Wu M. Liu H. Chen W. Fujimoto Y. Liu J. 2009 Hepatic expression of long-chain acyl-CoA synthetase 3 is upregulated in hyperlipidemic hamsters. Lipids. 44: 989–998. 19756806 10.1007/s11745-009-3341-3

[18] Wang Y. L. Guo W. Zang Y. Yancey G. C. Vallega G. Getty-Kaushik L. Pilch P. Kandror K. Corkey B. E. 2004 Acyl coenzyme A synthetase regulation: putative role in long-chain acyl coenzyme A partitioning. Obes. Res. 12: 1781–1788. 15601973 10.1038/oby.2004.221

[19] Wang D. Dubois R. N. 2012 Epoxyeicosatrienoic acids: a double-edged sword in cardiovascular diseases and cancer. J. Clin. Invest. 122: 19–22. 22182836 10.1172/JCI61453 PMC3248310

[20] Ou J. Tu H. Shan B. Luk A. DeBose-Boyd R. A. Bashmakov Y. Goldstein J. L. Brown M. S 2001 Unsaturated fatty acids inhibit transcription of the sterol regulatory element-binding protein-1c (SREBP-1c) gene by antagonizing ligand dependent activation of the LXR. Proc. Natl. Acad. Sci. USA. 98: 6027–6032. 11371634 10.1073/pnas.111138698 PMC33416

[21] Spector A. A. 2009 Arachidonic acid cytochrome P450 epoxygenase pathway. J. Lipid Res. 50(Suppl): S52–S56. 18952572 10.1194/jlr.R800038-JLR200 PMC2674692

[22] Askari B. Kanter J. E. Sherrid A. M. Golej D. L. Bender A. T. Liu J. Hsueh W. A. Beavo J. A. Coleman R. A. Bornfeldt K. E. 2007 Rosiglitazone inhibits acyl-CoA synthetase activity and fatty acid partitioning to diacylglycerol and triacylglycerol via a peroxisome proliferator-activated receptor-gamma-independent mechanism in human arterial smooth muscle cells and macrophages. Diabetes. 56: 1143–1152. 17259370 10.2337/db06-0267 PMC2819351

[23] Guijas C. Perez-Chacon G. Astudillo A. M. Rubio J. M. Gil-de-Gomez L. Balboa M. A. Balsinde J. 2012 Simultaneous activation of p38 and JNK by arachidonic acid stimulates the cytosolic phospholipase A2-dependent synthesis of lipid droplets in human monocytes. J. Lipid Res. 53: 2343–2354. 22949356 10.1194/jlr.M028423 PMC3466003

[24] Hershko A. Ciechanover A. 1992 The ubiquitin system for protein degradation. Annu. Rev. Biochem. 61: 761–807. 1323239 10.1146/annurev.bi.61.070192.003553

[25] Schoonjans K. Watanabe M. Suzuki H. Mahfoudi A. Krey G. Wahli W. Grimaldi P. Staels B. Yamamoto T. Auwerx J. 1995;. Induction of the acyl-coenzyme A synthetase gene by fibrates and fatty acids is mediated by a peroxisome proliferator response element in the C promoter. J. Biol. Chem. 270: 19269–19276. 7642600 10.1074/jbc.270.33.19269

[26] Achouri Y. Hegarty B. D. Allanic D. Becard D. Hainault I. Ferre P. Foufelle F. 2005 Long chain fatty acyl-CoA synthetase 5 expression is induced by insulin and glucose: involvement of sterol regulatory element-binding protein-1c. Biochimie. 87: 1149–1155. 16198472 10.1016/j.biochi.2005.04.015

[27] Cao A. Li H. Zhou Y. Wu M. Liu J. 2010 Long chain acyl-CoA synthetase-3 is a molecular target for peroxisome proliferator-activated receptor delta in HepG2 hepatoma cells. J. Biol. Chem. 285: 16664–16674. 20308079 10.1074/jbc.M110.112805 PMC2878065

[28] Weedon-Fekjaer M. S. Dalen K. T. Solaas K. Staff A. C. Duttaroy A. K. Nebb H. I. 2010 Activation of LXR increases acyl-CoA synthetase activity through direct regulation of ACSL3 in human placental trophoblast cells. J. Lipid Res. 51: 1886–1896. 20219900 10.1194/jlr.M004978 PMC2882745

[29] Kudo T. Tamagawa T. Kawashima M. Mito N. Shibata S. 2007 Attenuating effect of clock mutation on triglyceride contents in the ICR mouse liver under a high-fat diet. J. Biol. Rhythms. 22: 312–323. 17660448 10.1177/0748730407302625

[30] Orlando U. Cooke M. Cornejo M. F. Papadopoulos V. Podesta E. J. Maloberti P. 2013 Characterization of the mouse promoter region of the acyl-CoA synthetase 4 gene: role of Sp1 and CREB. Mol. Cell. Endocrinol. 369: 15–26. 23376217 10.1016/j.mce.2013.01.016

[31] Kaller M. Liffers S. T. Oeljeklaus S. Kuhlmann K. Roh S. Hoffmann R. Warscheid B. Hermeking H. 2011 Genome-wide characterization of miR-34a induced changes in protein and mRNA expression by a combined pulsed SILAC and microarray analysis. Mol. Cell. Proteomics. 10: M111.010462. 10.1074/mcp.M111.010462 PMC3149097 21566225

[32] Cao Y. Dave K. B. Doan T. P. Prescott S. M. 2001 Fatty acid CoA ligase 4 is up-regulated in colon adenocarcinoma. Cancer Res. 61: 8429–8434. 11731423

[33] Klett E. L. Chen S. Edin M. L. Li L. O. Ilkayeva O. Zeldin D. C. Newgard C. B. Coleman R. A. 2013 Diminished acyl-CoA synthetase isoform 4 activity in INS 832/13 cells reduces cellular epoxyeicosatrienoic acid levels and results in impaired glucose-stimulated insulin secretion. J. Biol. Chem. 288: 21618–21629. 23766516 10.1074/jbc.M113.481077 PMC3724621

