# Supplementary material for: Arachidonic acid downregulates acyl-CoA synthetase 4 expression by promoting its ubiquitination and proteasomal degradation
Source: J Lipid Res. 2014 Aug;55(8):1657–67. doi: 10.1194/jlr.M045971 (PMC4109760; doi:10.1194/jlr.M045971)
Supplement: Supplemental Data [file supp_55_8_1657__index.html]

Arachidonic acid down regulates acyl-CoA synthetase 4 expression by promoting its ubiquitination and proteasomal degradation — Arachidonic acid downregulates acyl-CoA synthetase 4 expression by promoting its ubiquitination and proteasomal degradation — Supplemental Data 

# Arachidonic acid downregulates acyl-CoA synthetase 4 expression by promoting its ubiquitination and proteasomal degradation

## Supplemental Data

**Files in this Data Supplement:**

- supplemental figure - contains three supplemental figures and two tables and legends
