# Supplementary material for: Arachidonic acid downregulates acyl-CoA synthetase 4 expression by promoting its ubiquitination and proteasomal degradation
Source: J Lipid Res. 2014 Aug;55(8):1657–67. doi: 10.1194/jlr.M045971 (PMC4109760; doi:10.1194/jlr.M045971)
Supplement: Supplemental Data [file supp_M045971_jlr.M045971-1.pdf]

Supplementary Figure I

A

```
                           10        20        30        40        50        60        70        80
Hamster ACSL4              MAKRIKAKPTSDKPGSPYRSVTHFDSLAVIDIPGADTLDKLFDHAVAKFGKKDSLGTREILSEENEMQPNGKVFKKLILG 80
Human ACSL4                MAKRIKAKPTSDKPGSPYRSVTHFDSLAVIDIPGADTLDKLFDHAVSKFGKKDSLGTREILSEENEMQPNGKVFKKLILG 80
Mouse ACSL4                MAKRIKAKPTSDKPGSPYRSVTHFDSLAVIDIPGADTLDKLFDHAVAKFGKKDSLGTREILSEENEMQPNGKVFKKLILG 80
Rat ACSL4                  MAKRIKAKPTSDKPGSPYRSVTHFDSLAVIDIPGADTLDKLFDHAVAKFGKKDSLGTREILSEENEMQPNGKVFKKLILG 80
anti human ACSL4 peptide   MAKRIKAKPTSDKPG                                                                  15
(Dr. Prescott)
```

B

```
                                     710       720
Hamster ACSL3                 KRKELKTHYQADIERMYGRK   721
Human ACSL3                   KRKELKTHYQADIERMYGRK   721
Mouse ACSL3                   KRKELKTHYQADIERMYGRK   721
Rat ACSL3                     KRKELKTHYQADIERMYGRK   721
anti-hamster ACSL3 peptide          THYQADIERMYGRK   14
```

C

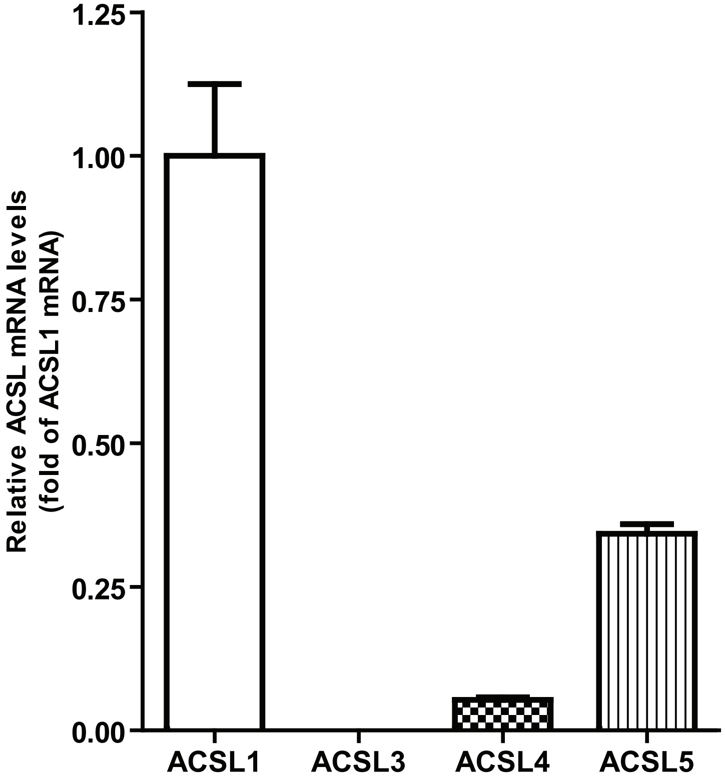

Supplementary Figure II

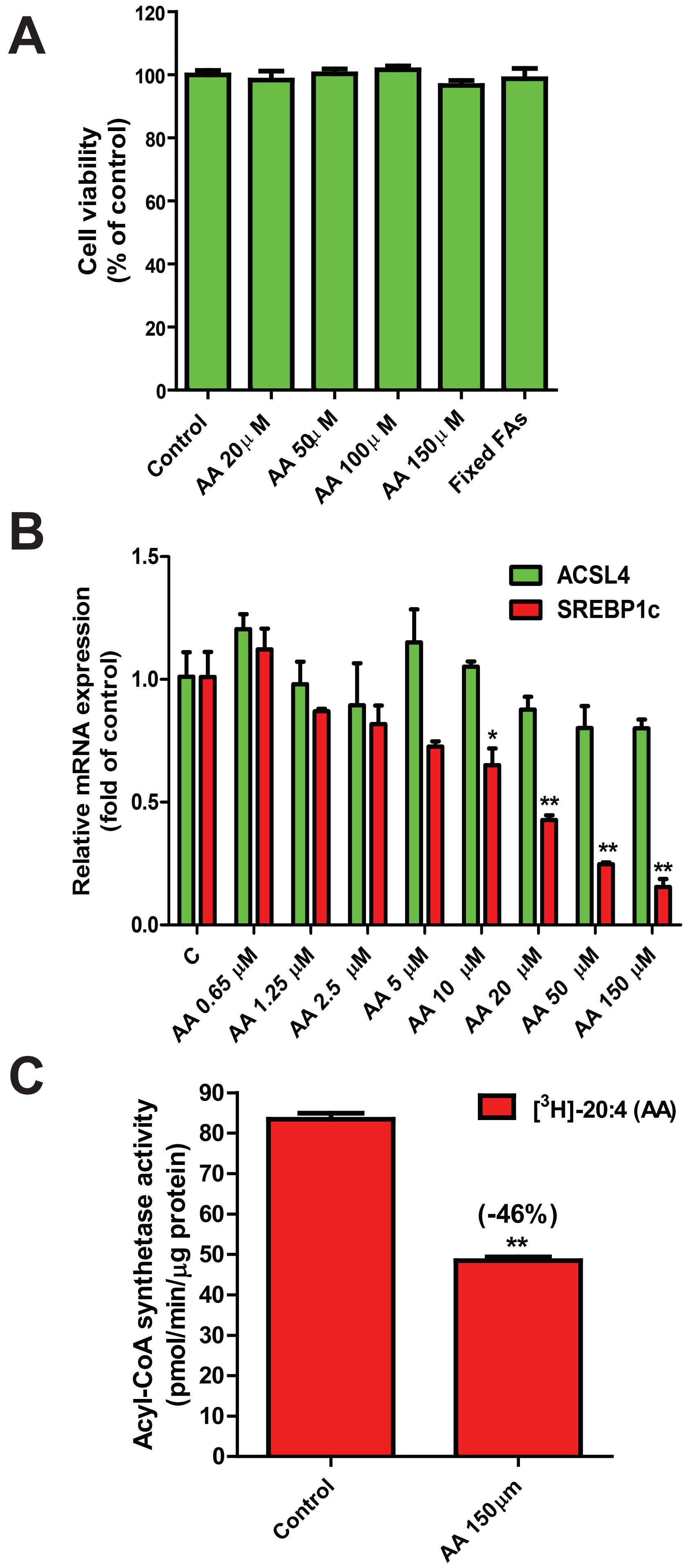

Supplementary Figure III

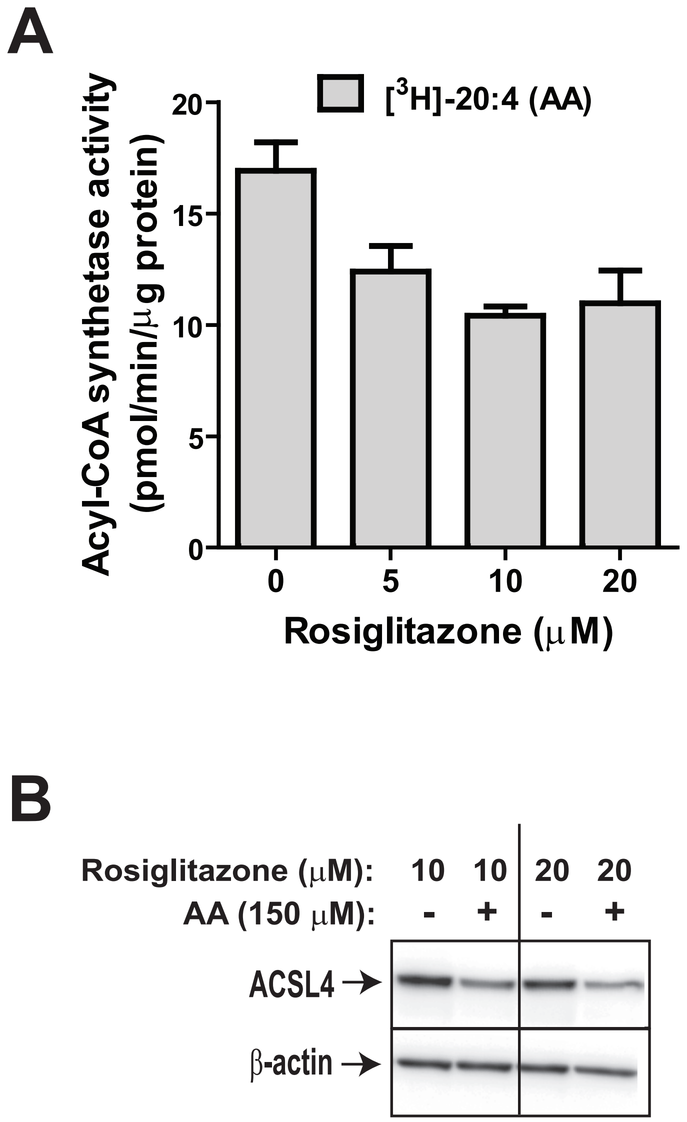

Supplementary Figure legends

Supplementary Fig. I

(A) Alignment of human, mouse, rat and hamster ACSL4 N-terminal sequences with antigen peptide sequence of anti-ACSL4 antibody.

(B) Alignment of human, mouse, rat and hamster ACSL3 C-terminal sequences with antigen peptide sequence of anti-ACSL3 antibody.

(C) qRT-PCR was conducted to determine relative mRNA levels of ACSL isoforms in liver samples of mice fed regular chow diet. After normalization with GAPDH mRNA levels, the mRNA level of ACSL1 was expressed as 1.

Supplementary Fig. II. Effects of AA treatment on cell viability, gene expression and ACSL enzyme activity.

(A) HepG2 cells were treated with indicated concentrations of AA or the mixture of FAs for 24 h and cell viability was measured using the commercial kit.

(B) HepG2 cells were treated with indicated concentrations of AA for 24 h before isolation of total RNA for gene expression analysis by qPCR.

(C) HepG2 cells were treated with 150 μM concentration of AA for 24 h and cell homogenates were isolated. Initial rates of total ACSL activity in cell homogenate were measured using 5 µg of cell homogenate at 37°C in the presence of [^{3}H] labeled AA.

Supplementary Fig. III. Rosiglitazone had no effects on AA-induced downregulation of ACSL4 protein levels.

(A) HepG2 cell lysates were incubated in the presence of [3H]-AA, ATP, CoA and indicated concentrations of rosiglitazone for 30 min at room temperature. Generated [^{3}H]-AA-CoA was separated, and the radioactivity was determined via a scintillation counter. The results are the representative means of picomoles arachidonoyl-CoA formed/minute ±SE, performed in triplicate.

(B) HepG2 cells were treated with 10 or 20 μM rosiglitazone in the absence or presence of 150 uM AA. Total cell lysates were isolated and 30 μg/lane was analyzed for ACSL4 protein levels by Western blot.

Supplementary Table I. Sequences of primers used in this study

Real Time Primers:

**Human ACSL1 Forward**	CTTCTGGTACGCCACGAGAC
**Human ACSL1 Reverse**	GTCGCTGTCAAGTAGTGCG
**Human ACSL3 Forward**	CCACGCCTGCGGCACATCAT
**Human ACSL3 Reverse**	TGGTTTTCCATGCTGGCCTTGG
**Human ACSL4 Forward**	CCCTGAAGGATTTGAGATTCACA
**Human ACSL4 Reverse**	CCTTAGGTCGGCCAGTAGAAC
**Human ACSL5 Forward**	GAGTACCTGGGTTCCTGTCTC
**Human ACSL5 Reverse**	AAAGATGCCGACAAACTGGTC
**Human GAPDH Forward**	ATGGGGAAGGTGAAGGTCG
**Human GAPDH Reverse**	GGGGTCATTGATGGCAACAATA
**Mouse ACSL1 Forward**	ATCTGGTGGAACGAGGCAAG
**Mouse ACSL1 Reverse**	TCCTTTGGGGTTGCCTGTAG
**Mouse ACSL3 Forward**	TCTTGCAAACAAAGCTGAAGGA
**Mouse ACSL3 Reverse**	GGTTGGAGGCTTCCCATCAA
**Mouse ACSL4 Forward**	CTTCCTCTTAAGGCCGGGAC
**Mouse ACSL4 Reverse**	TGCCATAGCGTTTTTCTTAGATTT
**Mouse ACSL5 Forward**	GGCCAAACAGAATGCACAGG
**Mouse ACSL5 Reverse**	GATGCAGATCTCGCCTTCGT
**Mouse GAPDH Forward**	ATGGTGAAGGTCGGTGTGAA
**Mouse GAPDH Reverse**	ACTGGAACATGTAGACCATGTAGT

Supporting Table II. Effects of inhibitors to various arachidonic acid (AA) metabolic pathways and signaling pathways on AA-mediated suppression of ACSL4 protein expression. HepG2 cells were treated with individual inhibitors at indicated concentrations for 1 h prior to the addition of FA-free BSA conjugated AA (150 μM) to the culture medium. After 24 h, cell lysates were prepared for Western blotting using anti-ACSL4 and anti-β-actin.

Gene	Compound name	Concentration used	Compound Function	Effect on AA-induced ACSL4 protein degradation
COX-1	SC-560	10 uM	A selective inhibitor of COX-1 activity	No effect
COX-2	CAY10404	10 uM	A highly selective inhibior of COX-2 activity	No effect
COX-1 and COX-2	Aspirin	10 uM	Inhibits bothe COX-1 and COX-2 activity	No effect
COX-1 and COX-2	Indomethacin	10 uM	Inhibits bothe COX-1 and COX-2 activity with greater COX-1 selectivity	No effect
5-Lipoxygenase	Zileuton	30 uM	5-Lipoxygenase specific inhibitor	No effect
12-Lipoxygenase and 5-Lipoxygenase	3,4-Dihydroxyphenyl Ethanol	100 uM	Inhibits 12-Lipoxygenase and 5-Lipoxygenase activity specifically	No effect
15-Lipoxygenase	15-Lipoxygenase Inhibitor 1	40 uM	Inhibits 15-Lipoxygenase activity specifically	No effect
12-Lipoxygenase, 5-Lipoxygenase and 15-Lipoxygenase	Nordihydroguaiaretic acid	100 uM	A potent antioxidant compound; 5-Lipoxygenase, 12-Lipoxygenase and 15-Lipoxygenase Inhibitor	No effect
CYP4A and CYP4F	HET0016	10 uM	A potent and selective inhibitor of HETE production by inhibitng CYP4A and CYP4F	No effect
CYP2C8	Gemfibrozil	400 uM	Lower High-cholesterol and Triglyceride Levels; Strong CYP2C8 Inhibitor	No effect
CYP2C9, CYP2C19 and CYP3A	Fluconazole	25 uM	Drug to treat serious fungal or yeast infections; A potent CYP2C9 inhibitor	No effect
CYP2J2	Danazol	100 uM	A weak androgen; anterior pituitary suppressant; reported specific CYP 2J2 Inhibitor	No effect
General PKC	Calphostin C	500 nM	Cell Permeable, highly specific general PKC inhibitor	No effect
PKC-α, PKC-β, PKC-ε	Ro-32-0432	100 nM	Cell Permeable, specific PKC inhibitor for PKC-α, PKC-β and PKC-ε	No effect
PKC-α, PKC-β, PKC-γ, PKC-ε	Bisindolylmaleimide 1	100 nM	High selective inhibitor for PKC-α, PKC-β, PKC-γ, PKC-ε	No effect
PKC-α, PKC-β	Go 6976	100 nM	Selectively inhibit PKC-α and PKC-β	No effect
General PKC	Chelerythrine Chloride	5 uM	Cell Permeable, highly specific general PKC inhibitor	No effect
PKA	H-89	10 uM	Specific PKA Inhibitor	No effect
PKA	8-Br-cAMP	1 mM	Long acting PKA activator	No effect
ACSL4	Rosiglitazone	10 uM	PPAR-γ agoinst; Selectively inhibits ACSL4	No effect
MEK1 and MEK2	U0126	10 uM	Highly selective inhibitor of both MEK1 and MEK2	No effect
p38α and p38β	SB203580	10 uM	Specific inhibitor of p38α and p38β	No effect
PI3Ks	LY294002	20 uM	Potent Inhibitor of PI3Ks	No effect
JNKs	SP600125	20 uM	Inhibitor of JNKs	No effect
GSK-3β	Lithium Chloride	20 mM	Lithium Chloride is an inhibitor of GSK-3β	No effect
AA metabolic products	PGE2	10 uM	Prostaglandin and product of the COX pathway; induce inflammation or act as vasodilator	No effect
